# Investigating the relationship between sleep disturbances and psychopathology In children and adolescents with microdeletion of 22q11 chromosome: an exploratory study

**DOI:** 10.3389/fpsyt.2025.1595492

**Published:** 2025-07-23

**Authors:** Maria Rosaria Lala, Domenica Bellantoni, Federica Alice Maria Montanaro, Maria Pontillo, Paolo Alfieri, Stefano Vicari

**Affiliations:** ^1^ Department of Life Sciences and Public Health, Università Cattolica Del Sacro Cuore, Rome, Italy; ^2^ Child & Adolescent Neuropsychiatry Unit, Bambino Gesù Children’s Hospital, IRCCS, Rome, Italy; ^3^ Department of Education, Psychology, Communication, University of Bari Aldo Moro, Bari, Italy

**Keywords:** behavioral problems, 22q11.2 deletion syndrome (22q11.2DS), cognitive function, adaptive functioning, psychiatric issues, sleep disorders, parental stress

## Abstract

**Introduction:**

22q11.2 deletion syndrome (22q11.2DS), also known as velo-cardiofacial syndrome or DiGeorge syndrome (DGS) is highly variable in phenotype, encompassing a wide range of physical and neuropsychiatric manifestations (ID, ADHD, ASD, anxiety, major depressive disorder,obsessive-compulsive disorder, schizophrenia). In this retrospective study, we aimed to assess the clinical significance of sleep disturbances and their relationship with functional impairment in a cohort of 52 children and adolescents with 22q11.2DS, as well as psychological distress in their parents. Standardized measures, including the Sleep Disturbance Scale for Children (SDSC) and the Parenting Stress Index-Short Form (PSI-SF), were administered to parents.

**Methods:**

The sample consisted of 26 males and 26 females, aged 5 to 18 years. Participants were referred to the Day Hospital follow up of Child and Adolescent Neuropsychiatry Unit in the Bambino Gesù Children’s Hospital in Rome, Italy, between January 2023 and December 2023. The total cohort was divided into two main groups based on the presence of sleep problems: (1) Group 1, with sleep problems, and (2) Group 2, without sleep problems. Both groups demonstrated low mean IQ scores and low general adaptive functioning, as measured by the Wechsler Intelligence Scale for Children (WISC-IV) and the ABAS II General Adaptive Composite (GAC), respectively. Furthermore, Group 1 exhibited significantly lower functioning when assessed using the CGAS. Additionally, Group 1 reported higher levels of self-reported anxiety symptoms (MASC-2) compared to Group 2. While none of the results reached the clinical range, scores in Group 1 were generally higher, particularly on the “Performance Fears” subscale. A similar trend was observed in the “Negative Self-Esteem” subtest of the CDI 2 (self-report form). Although the average questionnaire scores did not fall within the clinical range, KSADS psychiatric diagnoses revealed the presence of various psychiatric disorders. Unexpectedly, these disorders were more prevalent in the group without sleep problems, except for anxiety disorders, which showed similar prevalence across both groups. Regarding parental stress, as measured by the PSI-SF, we did not observe a significant relationship between sleep disorders and parental stress, on the contrary to our expectations.

**Results and discussion:**

Our study is one of the few to specifically investigate sleep problems in the pediatric population with 22q11.2DS and the first to use the Sleep Disturbance Scale for Children (SDSC) to assess various aspects of sleep disorders in this group. Further studies are required to draw more consistent conclusions.

## 1 Introduction

22q11.2 deletion syndrome (22q11.2DS), also known as velo-cardiofacial syndrome or DiGeorge syndrome (DGS), is a neurodevelopmental disorder resulting from a microdeletion on the long arm of chromosome 22. As one of the most common microdeletion syndromes, it has an estimated prevalence of 1 in 3,000–6,000 live births (1) The phenotype of 22q11.2DS is highly variable, encompassing a wide range of physical and neuropsychiatric manifestations. Physical features commonly observed in individuals with 22q11.2DS include hooded or swollen eyelids, a tubular nose, a broad nasal tip, a small mouth, hypertelorism, mild ear abnormalities (2) and palatal and velopharyngeal abnormalities, which may contribute to obstructive sleep apnea syndrome (OSAS) (3). Additional features of the syndrome include an absent or hypoplastic thymus, cardiac defects, hypocalcemia, and parathyroid hypoplasia. The thymic deficiency is particularly concerning, with its absence (found in less than 1% of patients) often leading to severe combined immunodeficiency (SCID) (4). Gastrointestinal (GI) symptoms, such as constipation and abdominal pain, are also prevalent, with emerging evidences suggesting a link between these symptoms and the psychological issues associated with 22q11.2DS (5). Leader et al. demonstrated a moderate positive correlation between GI symptoms and sleep problems, indicating a bidirectional relationship between these two comorbidities in children and adolescents with 22q11.2DS (5). The neuropsychiatric profile of 22q11.2DS typically includes intellectual disability (ID) and various neurodevelopmental disorders, such as attention deficit hyperactivity disorder (ADHD), autism spectrum disorder (ASD), anxiety, major depressive disorder and obsessive-compulsive disorder. Additionally, approximately one-third of individuals with 22q11.2DS develop psychotic disorders resembling schizophrenia (SCZ) (6, 7) and that’s why 22q11.2DS is considered a genetic model of psychosis. Many of these neuropsychiatric conditions are closely associated with sleep quality (8–11). Research has shown that higher rates of sleep disorders in children with 22q11.2DS correlate with ADHD symptoms, anxiety, impaired executive functioning, and coordination difficulties (11): up to 80% of individuals with both 22q11.2DS and a mental health disorder experience sleep disturbances, which in turn exacerbate psychiatric symptoms (12). Sleep disturbances, including obstructive sleep apnea (OSA), are common in individuals with 22q11.2DS (3). Given the high comorbidity between sleep disorders and psychosis, along with 22q11.2DS being considered a genetic model for SCZ, this syndrome presents a unique opportunity to explore the connection between these phenomena (6, 7). Despite its significance, there are very few studies examining sleep disorders specifically in individuals with 22q11.2DS. In 2018, for instance, Yirmiya et al. investigated the connection between sleep quality, inflammatory markers, and cognitive deficits in 22q11.2DS, with a study including thirty-three individuals with 22q11.2DS and twenty-four healthy controls. They found that individuals with 22q11.2DS had significantly poorer sleep quality, independently from the psychiatric/physical conditions usually associated to this genotype. The authors also depicted a strong connection between poor sleep quality and cognitive impairments, suggesting that sleep disturbances may partially explain cognitive deficits in 22q11.2DS (13). In 2021, Hyde et al. (14) published a study in which they examined the associations between subjective sleep and affect in thirty-one adults with 22q11DS compared to twenty-four healthy controls, finding that people with 22q11DS exhibited a longer sleep latency and a great number of night wakings, with no between differences with controls in subjective sleep quality (14). Individuals with 22q11DS reported clinical significant affective disturbances, accordingly to previous data reporting high degree of emotional and mood disorder in this population (6, 7, 15). In 2022, O’Hora et al. (16) performed an innovative study aiming to examine the role of copy number variation at the 22q11.2 locus in influencing the prevalence, severity, and psychiatric impact of sleep disturbances. They compared subjective sleep disturbances and their relationship to psychiatric symptoms in one-hundred and seven 22q11.2 deletion carriers, forty-two 22q11.2 duplication (22qDup) carriers, and eighty-eight age- and sex-matched controls over a one-year period. Both 22qDel and 22qDup carriers reported poorer sleep compared to controls, although no significant differences were observed between the two carrier groups. Poor sleepers exhibited higher scores for psychosis risk, anxiety, depression, somatic complaints, thought problems, aggressive behavior, and ASD symptoms. Notably, the difference between subdomains of the Child Behavior Checklist (CBCL) (17) for good and poor sleepers was more pronounced in 22qDel carriers than in 22qDup carriers (16). In 2023, Reich et al. (7) performed the first longitudinal study examining sleep disorders in sixty-nine individuals with 22q11DS and thirty-eight healthy controls with a follow up evaluation after around 3 years. They examined the relationship between different measures of sleep disorders and clinical vulnerability to psychosis, measured by the Structured Interview for Psychosis-risk Syndromes (SIPS) (18). In their study, they showed that disrupted sleep strongly predicts longitudinal clinical trajectory towards psychosis in 22q11DS, even though the correlation between sleep disorders and other psychopathological measures was not assessed (7). While the previous findings help to understand the relationship between sleep and psychosis risk and between sleep disorders and affective issues in people with 22q11DS, no studies have examined the broader impact of sleep disturbances on parental distress in this population, although the impact of sleep disturbances on parents’ quality of life is a critical consideration. Studies of families with children experiencing neurodevelopmental disorders or disabilities (e.g., Down syndrome, cerebral palsy, ID) have consistently reported higher parental stress and lower quality of life compared to parents of typically developing children (19–29). Given the strong correlation between sleep disturbances, mental illness, and neurodevelopmental disorders, it is reasonable to hypothesize that parents of children with 22q11.2DS and sleep disturbances may experience higher stress and a lower quality of life, potentially exacerbating their child’s psychopathology through parenting practices.

In this context, the primary aim of the present study was to assess the clinical significance of sleep disturbances and their relationship with functional impairment in a cohort of 51 children and adolescents with 22q11.2DS, as well as psychological distress in their parents. Standardized measures, such as the Sleep Disturbance Scale for Children (SDSC) and the Parenting Stress Index Short Form (PSI-SF), were administered.

## 2 Materials and method

### 2.1 Participants

In this retrospective study fifty-two children and adolescents with 22q11DS were recruited, referred to the Day Hospital follow up of Child and Adolescent Neuropsychiatry Unit in the Bambino Gesù Children’s Hospital in Rome (Italy). Specifically, the retrospective data were acquired from records extracted from a dedicated database collecting pseudo-anonymized data, created for medical practice and refer to a period between January 2023 and December 2023. All the children were in follow up for their genetic condition, by 6 months checks up, about the cognitive and psychiatric risk factors. They were subject to cognitive- behavioral therapy and they took neither drug nor melatonin integration.

Inclusion criteria were, besides the diagnosis of microdeletion of 22q11 chromosome on the analysis of the karyotype, the age ranging between 5 and 18 years. Exclusion criteria consisted of clinical suspect of neurological conditions and language barrier hampering questionnaire compilation by parents. After controlling for these criteria, fifty-two children, twenty-six males and twenty-six females aged from 5 to 18 years, with a total mean age of 14.23 years, were included in the study. The final sample was then divided into two main groups aligned by sleep problems: 1) Group 1 with sleep problems and 2) Group 2 without sleep problems, as depicted by the total score of the SDSC (Screening for Sleep Disorders, *see paragraph 2.2.3*). Both groups demonstrated low mean IQ scores and general adaptive functioning, as measured by the Wechsler Intelligence Scale for Children (WISC-IV) and ABAS II General Adaptive Composite (GAC), respectively, with results falling within the clinical range. Demographic data are summarized in **Table 1** (*All descriptive statistics regarding IQ, as assessed by the Wechsler scales, and adaptive functioning, measured by the ABAS II, can be consulted in* **Supplementary Table 1S**).

**Table 1:** Demographic characteristics.

Group	Age (Mean)	N	Gender	WISC-IV IQ Mean	ABAS GAC Mean
Group 1	14.29	16	10 Male 6 Female	72.5	71.75
Group 2	14.12	36	16 Male 20 Female	68.9	76.33

Group 1, participants with sleep problems; Group 2, participants without sleep problems; N, number of subjects in each group; IQ, intelligent quotient measured by WISC-IV; M, mean; GAC, ABAS II general adaptive composite.

The study was conducted in accordance with the Declaration of Helsinki and in accordance with Italian legal and ethical requirements for clinical data. IRB approval was obtained for the data reporting in the present report (Ethics Committee of Bambino Gesù Children’s Hospital practice number 3161/2023). Ethical approval date: 27 June 2023.

### 2.2 Measures

#### 2.2.1 Cognitive assessment

Cognitive assessment was performed through the administration of the Wechsler Intelligence Scale for Children (WISC-IV).This is a four-factor intelligence battery for children aged between 6 and 16 years of age. This battery is comprised of 10 core subtests, which combine to form four psychometrically validated factor scores. The Verbal Comprehension Index (VCI) includes the Similarities, Vocabulary, and Comprehension subtests; the Perceptual Reasoning Index (PRI) includes the Block Design, Picture Concepts, and Matrix Reasoning subtests; the Working Memory Index (WMI) includes the Digit Span and Letter-Number Sequencing subtests; and the PSI includes the Coding and Symbol Search subtests. All 10 subtests combine to form a full-scale IQ (FSIQ) score. Supplemental subtests were not included in this study as not all children completed them and they are not required to calculate index and FSIQ scores. WISC-IV index standard scores have a mean of 100 and a standard deviation of 15 whereas subtest scores have a mean of 10 and a standard deviation of 3 (30).

#### 2.2.2 Evaluation of adaptive abilities

To assess the presence of impairments in adaptive behaviors, necessary for socialization, communication, and daily functioning, we used the Adaptive Behavior Assessment System II (ABAS-II), a parent/primary caregiver questionnaire. ABAS-II consists of eleven skill areas organized into three general domains: conceptual, practical, and social. The composite and domain scores are standard scores with a norm-referenced mean of 100 and a standard deviation of 15 (31)

#### 2.2.3 Screening for sleep disorders

Sleep disturbances were assessed by means of the Sleep Disturbance Scale for Children (SDSC), a questionnaire that has demonstrated through validation an adequate level of internal consistency, test-retest reliability, and availability of normative data (32–34) The SDSC explores the presence of sleep disorders during the previous six months and contains 26 items with Likert scale values of 1–5. The questionnaire consists of 26 items subdivided into six sleep disorder subscales: disorders in initiating and maintaining sleep (DIMS), sleep breathing disorders (SBD),disorders of arousal (DA), sleep/wake transition disorders (SWTD), disorders of excessive somnolence (DOES), and sleep hyperhidrosis (SHY). The SDSC total score has demonstrated suitable concurrent validity with diagnosed sleep disorders (insomnia, hypersomnia, respiratory disturbances during sleep, and parasomnias). The subscales have demonstrated low inter-correlations. The sum of scores provides a total sleep score with a possible range from 26 to 130; a T score major than 70 was regarded as pathological. Results are classified into pathological, borderline, or normal, using a cut off value according to the validation criteria of the test. Due to a different prevalence of sleep disturbances in younger children, for the age range considered in the current study (3–6 years old), it has been proposed a different factorial structure from the original SDSC (33) The most common areas of sleep disturbance in preschoolers are divided into six factors: difficulty in initiating (DIS) and maintaining sleep (DMS), sleep-disordered breathing (SDB), parasomnias (PAR), disorders of excessive somnolence (DOES), non-restorative sleep (NRS), and sleep hyperhidrosis (SHY) (33).

#### 2.2.4 Psychopathological assessment

The psychopathological assessment was conducted through the administration of the following tests:

- Conners’ Rating Scales Long Version Revised (CPRS).
  This 80-items questionnaire is completed by parents to obtain a measure of attention deficit and hyperactivity disorder criteria for hyperactivity and inattention. The questionnaire contains scales from A to N (A oppositional; B inattention; C hyperactivity; D anxiety; E perfectionism; F social problems; G psychosomatic problems; H ADHD index; I CGI: restlessness; J CGI: emotional instability; K CGI: total; L DSM-IV: inattention; M, DSM-IV: hyperactivity/impulsivity; N DSM-IV: total). Scores can be finally converted to a T score (35).
- The Kaufman’s Schedule for Affective Disorders and Schizophrenia Present and Lifetime Version (K-SADS-PL) DSM-5 investigates the possible presence of psychopathological disorders according to DSM-5 (including schizophrenia spectrum disorders). The K-SADS-PL DSM-5, as proposed in the instrument’s manual by Kaufman et al., provides as a source of information not only according to the child/adolescent but also to the parents. In addition, for some particular cases (i.e., ID), the parent is considered the main source of information with respect to the child (36).
- The Children’s Depression Inventory (CDI) is used to assess depressive symptoms in children and adolescents, though it also includes items aimed to evaluating scholastic and relational concerns. It is composed of 27 items, which are ranked from 0 to 2, providing a total score in the range of 0–54. Symptoms are clustered into three subscales (1): negative mood (2), negative self-esteem, and (3) interpersonal problems. Participants answer to the questions about how they have felt over the past 2 weeks. The measure showed high internal consistency (α = 0.80) and significant correlations between item and total product moment (37)
- The Multidimensional Anxiety Scale for Children 2nd edition (MASC-2) is a 39-item self-report instrument for assessing anxiety symptoms in children and adolescents. The MASC-2 evaluates a broad spectrum of anxiety-related symptoms, including emotional, physical, cognitive, and behavioral aspects, across six scales and four subscales. It offers a comprehensive measure of both the extent and severity of anxiety symptoms. The responses are used to generate 11 T-scores (mean = 50, standard deviation = 10), which include: Total Score, Separation Anxiety/Phobias, Generalized Anxiety Disorder Index, Social Anxiety (Total, Humiliation/Rejection, Performance Fears), Obsessions & Compulsions, Physical Symptoms (Total, Tense/Restless, Panic), and Harm Avoidance. A total T-score of 60 or higher indicates an increased likelihood of at least one anxiety disorder. Items are clustered into four subscales (1): physical symptoms (2), social anxiety (3), harm avoidance, and (4) separation anxiety. This measure showed a good internal consistency (α = 0.60 to α = 0.85) and a high test–retest reliability (r = 0.79 to r = 0.93) (68–70) (38)
- The Children’s Global Assessment Scale (CGAS) is a measure used by clinicians to assess functioning in children and adolescents. It provides a total score for the level of disturbance in general functioning in the range of 0–100, with higher scores corresponding to higher levels of functioning. Studies demonstrated a fair to adequate inter-rate reliability (r = 0.53 to 0.87) (39)

#### 2.2.5 Parents’ psychopathological distress clinical assessment

Caregiver completed the Parenting Stress Index-Short Form (PSI-SF), a self-report questionnaire to investigate perceived parental distress, examining personal factors, parent–child interaction, and behavioral characteristics of the child. The PSI-SF consists of 36 questions, which capture three domains: parental distress, parent–child dysfunctional interaction, and difficult child. The Parental Distress (PD) subscale evaluates aspects that may affect parenting activities, such as limited social support and parental conflict with the partner. The Difficult Child (DC) subscale assesses parenting challenges related to a child’s self-regulation or behavioral difficulties. The Parent–Child Dysfunctional Interaction (P-CDI) subscale is created to assess unsatisfactory parent–child interactions. The sum of all questions results in the Total Stress score (40).

### 2.3 Statistical analysis

All statistical analyses were performed using JAMOVI software version 2.3.26.0. Descriptive analyses were performed to characterize groups of participants. Independent two-sample t test was carried out to compare differences between groups (group 1= sleep problems; group 2= no sleep problems) in all outcome measures. Uncontrolled effect sizes (Cohen’s d+) were calculated. We did not apply multiple-comparison correction to avoid the risk of type II errors, since the sample size is very small (VanderWeele & Mathur, 2019). We also conducted Chi-square analyses (binomial tests) to investigate whether the frequency of the DSM-5 main diagnostic classes was the same or different between the two patients groups. P < 0.05 was considered statistically significant.

## 3 Results

### 3.1 Descriptive analyses

All descriptive statistics are reported in **Supplementary Table 1S** (M, mean; SE, standard error). Analysis of the MASC 2 self-report revealed no statistically significant findings; the only score approaching significance was observed in the “Performance Fears” subscale for Group 1 (M = 61.9). Conversely, when analyzing the MASC 2 parent-report, scores in the at-risk range were identified for both groups across the following subscales: Total (Group 1: M = 62.1; Group 2: M = 61.2), Generalized Anxiety Disorder (GAD) (Group 1: M = 66; Group 2: M = 64.1), Social Anxiety (Group 1: M = 69.4; Group 2: M = 63.7), and Performance Fears (Group 1: M = 65.4; Group 2: M = 65.6). The Physical Symptoms-Total subscale showed a borderline score only in Group 1 (M = 61.7).

Regarding the CPRS subscales, borderline scores were found in both groups for Inattention (Group 1: M = 68.5; Group 2: M = 64), Anxiety (Group 1: M = 63.4; Group 2: M = 61), Social Problems (Group 1: M = 69.6; Group 2: M = 65.3), Psychosomatic Problems (Group 1: M = 61.8; Group 2: M = 61), ADHD (Group 1: M = 64; Group 2: M = 62.8), and DSM-IV Inattention (Group 1: M = 65.7; Group 2: M = 61.4). A borderline score was identified for Group 1 in the CPRS-DSM-IV Total scale (M = 62.4).

Analysis of the PSI did not reveal significant scores in questionnaires completed by fathers, even if borderline scores were found in the P-CDI (M = 63.6) and P-DC (M = 62.5) subscales of the questionnaires completed by mothers of patients in Group 1.

Examination of the SDSC subscales showed no significant scores for either group in the “Parasomnias” (PAR) (Group 1: M = 59.9; Group 2: M = 48.3) and “Sleep Hyperhidrosis” (SHY) (Group 1: M = 51.1; Group 2: M = 47.9) subscales. Borderline scores were noted only for Group 1 in the susbcale “Disorders of Maintaining Sleep” (DMS) (M = 64), “Sleep-Disordered Breathing” (SDB) (M = 62.6), and “Disorders of Excessive Somnolence” (DOES) (M = 66.4) subscales. Clinically significant mean scores were found solely in the subscale “Disorders of Initiating Sleep” (DIS) for Group 1 (M = 74.7).

### 3.2 Differences between groups in the questionnaire scores

Given the number of subtests in each questionnaire, only statistically significant comparisons are presented (**Table 2**). The dataset with all comparative analyses can be consulted in **Supplementary Material** (**Supplementary Table 2S**).

**Table 2:** Independent two-sample *t* test analyses.

Outcome measure	Group		t	df	p	Cohen’s *d*
	1 Mean	2 Mean				
MASC2 Self GAD	56.81	50.97	2.54	50	0.014*	0.76
MASC2 Self Social Anxiety	58.13	52.11	2.92	50	0.005**	0.88
MASC2 Self Humiliation/Rejection	53.44	48.44	2.62	50	0.012*	0.79
MASC2 Self Performance Fears	61.94	56.33	2.36	50	0.022*	0.70
MASC2 Self Physical Symptoms	57.63	51.27	2.72	50	0.009**	0.81
MASC2 Self Panic	54.75	51.27	2.28	50	0.027*	0.68
MASC2 Self Tense/Restless	58.50	51.86	2.77	50	0.008**	0.83
MASC2 Self Total Score	59.00	52.86	2.43	50	0.018*	0.73
CDI 2 Self Negative Self-Esteem	50.44	46.83	2.13	50	0.038*	0.64
cGAS	54.38	56.861	-2.33	50	0.023*	-0.70

Group 1= sleep problems; Group 2 = without sleep problems; M, mean; * = statistical strength (**p < 0.05; *p < 0.01).

Using t-tests, it was found that participants with sleep disturbances (Group 1) exhibited significantly higher levels of anxiety symptoms compared to those without sleep disturbances (Group 2). The p values and Cohen’s d effect size values derived from the MASC2-Self Report subtests indicated a moderate to high degree of significance in the differences between the subgroups across various subscales. A statistically significant difference was also observed in the “Negative Self-Esteem” subscale of the CDI-2 (t (50) = 2.13, p = 0.038, d = 0.64), indicating that individuals with sleep problems are more prone to experience low self-esteem and feelings of being unloved. Additionally, a significant difference between groups was identified in the c-GAS score (t (50) = -2.33, p = 0.023, d = 0.70), suggesting that, under identical genetic conditions, individuals with sleep disturbances exhibit poorer functioning compared to those without sleep problems, despite both groups experiencing similarly high levels of impairment (within the same score range). No significant differences were found in the PSI-SF and CPRS scores.

### 3.3 Descriptive analysis of the psychiatric diagnoses in the two groups and differences between groups in the frequency of psychiatric disorders

To assess the presence of psychiatric issues KSADS-PL was administered to patients and/or to their parents. Even if the results didn’t reach the statistical significance, we observed the presence of various psychiatric disorders in the both groups: depressive disorder (Group1:M= 1.94;Group 2 M=1.97), manic episode (Group 1M=2.0; Group2 M=2.0), psychotic episode (Group1:M= 1.94;Group 2 M=1.972), panic attack disease (Group 1M=2.0; Group2 M=2.0), separation anxiety (Group 1M=2.06; Group2 M=1.972), social phobia (Group 1M=2.06; Group2 M=1.944), specific phobias (Group 1M=2.06; Group2 M=1.972), generalized anxiety disorder (Group1:M= 1.94;Group 2 M=1.806), obsessive compulsive disorder (Group 1M=2.00; Group2 M=1.972), enuresis (Group 1M=2.0; Group2 M=2.0),encopresis (Group 1M=2.0; Group2 M=2.0), nervous anorexia (Group 1M=2.0; Group2 M=2.0), nervous bulimia (Group 1M=2.0; Group2 M=2.0), ADHD (Group 1M=1.69; Group2 M=1.861), oppositional defiant disorder (Group 1M=1.88; Group2 M=2.0), conduct disorder (Group 1M=2.0; Group2 M=2.0), tic disorder (Group 1M=2.0; Group2 M=2.0), substance abuse (Group 1M=2.0; Group2 M=2.0), post-traumatic stress disorder (Group 1M=2.0; Group2 M=2.0), disrupted mood dysregulation disorder (Group 1M=2.06; Group2 M=2.0), agoraphobia (Group 1M=2.0; Group2 M=2.0), selective mutism (Group 1M=2.0; Group2 M=2.0),autism spectrum disorder (Group 1M=2.0; Group2 M=2.0).

To investigate whether the two groups significantly differed in the prevalence of the most common mental disorders, we categorized psychiatric disorders into the following main subcategories: mood disorders (e.g., depression, bipolar disorder), anxiety disorders (e.g., separation, social, generalized anxiety), behavioral disorders (including oppositional defiant disorder, conduct disorder, and substance abuse), eating disorders, neurodevelopmental disorders (e.g., ADHD, Tic disorder), thought disorders (e.g., psychosis, obsessive-compulsive disorder), and enuresis/encopresis *(as shown in* **Supplementary Table 3S** *)*. We then performed Chi-square analyses (binomial tests) to identify differences. As shown in **Table 3**, we observed a higher frequency of psychiatric disorders in the group without sleep problems, except for anxiety disorders, which had a similar prevalence between the groups. Interestingly, eating disorders, neurodevelopmental disorders, and enuresis/encopresis were diagnosed only in the group with normal sleep patterns.

**Table 3:** Group differences in the frequencies of psychiatric diagnoses between groups.

	Group	Frequency	Tot	Prop	p
Mood	1	3	52	0.058	< .001
	2	49		0.942	< .001
Anxiety	1	21	52	0.404	0.212
	2	31		0.596	0.212
Behavioral	1	14	52	0.269	0.001
	2	38		0.731	0.001
Eating	1 2	0 52	52	1.000	< .001
Neurodevelopmental	1 2	0 52	52	1.000	< .001
Psychosis/OCD	1	7	52	0.135	< .001
	2	45		0.865	< .001
Enco/Enur	1 2	0 52	52	1.000	< .001

Group 1= sleep problems; Group 2 = without sleep problems.

## 4 Discussion

Our study is one of the few to specifically investigate sleep problems in the pediatric population with 22q11.2DS and the first to use the Sleep Disturbance Scale for Children (SDSC), in order to assess various aspects of sleep disorders in this group (32–34).

We recruited fifty-two children and adolescents with 22q11.2DS, dividing them into two main groups: one exhibiting sleep problems (Group 1) and one without sleep problems (Group 2), as assessed by the SDSC completed by parents. These results are different respect previous studies on this population, in which sleep disturbances were very common, overcoming the rate in general population. One study involving 140 young individuals with 22q11.2 syndrome found that 60% experienced sleep problems, compared to 40% without sleep disturbances and to 23% of healthy controls. The most common issues included restless sleep and insomnia, both of which were associated with high levels of anxiety, ADHD, and behavioral disorders (6). Another study involving 100 children with 22q11.2 syndrome found that 85% of them had clinically significant sleep disturbances, including difficulty initiating or maintaining sleep, frequent night-time awakenings, and resistance to falling asleep. These disturbances were linked to daytime behavioral problems and language delays (7). Other recent studies have shown that between 60% and 97% of patients with 22q11.2DS experience clinically significant sleep disorders, with prevalence varying depending on the diagnostic criteria and evaluation methods (16). As better explained below (where we enounce the limitations of the study) our cohort is relatively small, limiting the generalizability of the results to the entire population of children with 22q11.2DS, even if we selected participants matched for all other sociodemographic variables. The unequal size of the two groups, with children experiencing sleep problems significantly fewer than those without, may have reduced the statistical power of the analyses and limited the ability to generalize the findings.

We then compared the two groups using different standardized clinical measures.

The results can be summarized as follows:

- first- both groups exhibited low levels of cognitive and adaptive functioning, falling within the range of mild intellectual disability (ID), with no significant differences between the groups in IQ or General Adaptive Composite (GAC) scores. The presence of cognitive deficits was expected and aligns with the cognitive phenotype of 22q11.2DS (41). Regarding the differences between groups on standardized measures, we observed that the group with sleep problems exhibited higher levels of self-reported anxiety symptoms (MASC-2) compared to those without sleep disturbances. More specifically, while none of the results fell within the clinical range, scores in Group 1 were generally higher, particularly on the “Performance Fears” subscale, consistent with previous data linking sleep disturbances and anxiety in individuals with 22q11.2DS (42). A similar trend was observed in the “Negative Self-Esteem” subtest of the CDI 2 (self-report form), suggesting that children and adolescents in Group 1 may have lower self-esteem than those in Group 2. This finding aligns with prior research on the relationship between self-esteem, sleep quality, and cognitive performance in children with typical neurodevelopment (43). It is worth noting that while the average scores on clinical questionnaires did not fall within the clinical range, and the differences between groups were minimal, Group 1 exhibited significantly lower functioning (as measured by the CGAS) compared to Group 2, despite both groups scoring within the same range on other measures. This discrepancy may be explained in two ways: first, individuals with 22q11.2DS in our sample may have struggled to recognize or self-report their symptoms, leading to an underestimation of psychological difficulties on standardized questionnaires. Second, the cognitive-behavioral phenotype itself—rather than associated conditions like sleep disorders—may have a greater impact on individuals’ functioning. This explanation aligns with previous studies on 22q11.2DS and may account for the lack of significant differences between the groups classified based on sleep disorders (44). In line with the first explanation, it is important to note that while the average questionnaire scores did not fall within the clinical range, KSADS psychiatric diagnoses made by clinicians revealed the of various psychiatric disorders. This finding is consistent with previous research showing a higher prevalence of mental disorders in individuals with ID compared to the general population (44), as well as elevated rates of psychopathology in individuals with 22q11.2DS (45, 46). Interestingly, and somewhat unexpectedly, psychiatric disorders appeared to be more prevalent in the group without sleep problems, with the exception of anxiety disorders, which showed a similar prevalence across both groups. This can be explained in two ways: on one hand, these findings suggest that sleep problems alone may not predict psychiatric outcomes, partially contradicting previous research (6, 7, 14) which identified a strong correlation between sleep disturbances and psychological issues. On the other hand, it is possible that when more severe psychological conditions are present, both parents and patients may deprioritize or overlook sleep problems, which are often underestimated and not widely recognized as a separate clinical disorder by the general population. This highlights the importance of using structured interviews conducted by an expert clinician, in addition to self- or parent-report questionnaires, when assessing individuals with 22q11.2DS, particularly children, adolescents, and those with cognitive deficits. Taking into account the PSI-SF completed by parents, we did not observe any relationship between sleep disorders and parental stress, on the contrary to expectations. It is important to note that no clinical scores were reported in the questionnaires completed by either mothers or fathers in either group. This result contrasts with previous findings showing higher levels of parental stress in mothers of children with 22q11.2DS compared to typically developing children (47) and a stronger relationship between childhood psychopathology and parental stress (48). One possible explanation, consistent with previous research (49–51), is that cognitive impairment itself has a more significant impact on parental distress. Since the study population exhibited a cognitive profile slightly below the normative range, perceived stress levels may have been lower. This suggests that the severity of cognitive challenges could have a more pronounced effect on parental stress than other factors.

Further researches are needed to explore the relationship between neurodevelopmental disorders, psychopathology (including sleep disorders), and parental well-being, with ID as a potential mediator. Before drawing conclusions, it is important to acknowledge several significant limitations of this study:

1. the study included a relatively small cohort, which limits the generalizability of the results to the entire population of children with 22q11.2DS. Precautionally we aimed to minimize biases by selecting a sample of children and adolescents that could be divided into two groups based on the presence or absence of sleep disorders, ensuring that participants were matched for all other sociodemographic variables.
2. the unequal size of the two groups, with children experiencing sleep problems being significantly fewer than those without, may have reduced the statistical power of the analyses and limited the ability to generalize the findings. Additionally, it should be noted that our research was conducted in a clinical setting, specifically a research hospital where patients are referred for clinical issues rather than research purposes, which posed challenges in applying strict inclusion and exclusion criteria. 3. We used subjective assessment methods (e.g., self-report questionnaires) instead of direct evaluations of sleep problems, other psychological issues, and parental stress, which may have led to an underestimation of potential difficulties, partly due to challenges in completing the questionnaires or a lack of insight from the participants.

Further studies, including direct assessments, are therefore necessary.

## 5 Conclusions

In conclusion, our study is consistent with previous research highlighting the high rates of psychopathology and low cognitive/adaptive functioning in children and adolescents with 22q11.2DS. Furthermore, we identified a relationship between anxiety and sleep disorders in this population, although the small sample size limits our ability to draw definitive conclusions. To gain a more comprehensive understanding of the role of sleep disorders in the connection between 22q11.2DS and psychopathology, larger longitudinal studies incorporating both direct and indirect measures are necessary. Clinicians should be mindful of the critical role sleep quality plays in neurodevelopmental disorders, where the risk for psychopathology is notably higher than in the general population (44). Sleep disorder assessments should therefore be routinely included in clinical evaluation of these individuals.

## Data Availability

The original contributions presented in the study are included in the article/**Supplementary Material**. Further inquiries can be directed to the corresponding author.
