# Supplementary material for: Investigating the relationship between sleep disturbances and psychopathology In children and adolescents with microdeletion of 22q11 chromosome: an exploratory study
Source: Front Psychiatry. 2025 Jul 23;16:1595492. doi: 10.3389/fpsyt.2025.1595492 (PMC12325377; doi:10.3389/fpsyt.2025.1595492)
Supplement: Supplementary file 1 [file Table1.docx]

| **Table 1 S. Descriptive analysis** | | | | | | | | | | | | | |
| --- | --- | --- | --- | --- | --- | --- | --- | --- | --- | --- | --- | --- | --- |
|  | | **Group** | | **N** | | **Mean** | |  | |  | | **SE** | |
| FSQI |  | 1 |  | 16 |  | 72.50 |  |  |  |  |  | 4.9497 |  |
|  | | 2 |  | 36 |  | 68.944 |  |  |  |  |  | 3.9587 |  |
| WISC-IV VCI |  | 1 |  | 16 |  | 75.13 |  |  |  |  |  | 3.8771 |  |
|  | | 2 |  | 36 |  | 73.833 |  |  |  |  |  | 2.5466 |  |
| WISC-IV PRI |  | 1 |  | 16 |  | 72.44 |  |  |  |  |  | 2.3926 |  |
|  | | 2 |  | 36 |  | 72.889 |  |  |  |  |  | 2.9162 |  |
| WISC-IV WMI |  | 1 |  | 16 |  | 73.94 |  |  |  |  |  | 3.1431 |  |
|  | | 2 |  | 36 |  | 72.111 |  |  |  |  |  | 2.1635 |  |
| WISC IV PSI |  | 1 |  | 16 |  | 71.19 |  |  |  |  |  | 3.1480 |  |
|  | | 2 |  | 36 |  | 71.750 |  |  |  |  |  | 2.5815 |  |
| ABAS-GAC comp |  | 1 |  | 16 |  | 71.75 |  |  |  |  |  | 4.6766 |  |
|  | | 2 |  | 36 |  | 76.333 |  |  |  |  |  | 3.1414 |  |
| ABAS-DAC comp |  | 1 |  | 16 |  | 74.31 |  |  |  |  |  | 4.0494 |  |
|  | | 2 |  | 36 |  | 80.722 |  |  |  |  |  | 2.9656 |  |
| ABAS-DAS comp |  | 1 |  | 16 |  | 75.56 |  |  |  |  |  | 4.8459 |  |
|  | | 2 |  | 36 |  | 73.750 |  |  |  |  |  | 3.5611 |  |
| ABAS-DAP comp |  | 1 |  | 16 |  | 74.94 |  |  |  |  |  | 4.4558 |  |
|  | | 2 |  | 36 |  | 79.722 |  |  |  |  |  | 3.0388 |  |
| ABAS-GAC perc |  | 1 |  | 16 |  | 14.19 |  |  |  |  |  | 4.3962 |  |
|  | | 2 |  | 36 |  | 20.556 |  |  |  |  |  | 4.5610 |  |
| ABAS-DAC perc |  | 1 |  | 16 |  | 13.94 |  |  |  |  |  | 4.3641 |  |
|  | | 2 |  | 36 |  | 22.306 |  |  |  |  |  | 4.3794 |  |
| ABAS-DAS perc |  | 1 |  | 16 |  | 18.50 |  |  |  |  |  | 6.4537 |  |
|  | | 2 |  | 36 |  | 18.417 |  |  |  |  |  | 4.1078 |  |
| ABAS-DAP perc |  | 1 |  | 16 |  | 14.94 |  |  |  |  |  | 4.1558 |  |
|  | | 2 |  | 36 |  | 20.617 |  |  |  |  |  | 4.3737 |  |
| MASC2 Self Tot |  | 1 |  | 16 |  | 59.00 |  |  |  |  |  | 1.9979 |  |
|  | | 2 |  | 36 |  | 52.861 |  |  |  |  |  | 1.4244 |  |
| MASC2 Self sep anxiety/phobias |  | 1 |  | 16 |  | 59.94 |  |  |  |  |  | 1.5558 |  |
|  | | 2 |  | 36 |  | 55.333 |  |  |  |  |  | 1.4265 |  |
| MASC2 Self GAD |  | 1 |  | 16 |  | 56.81 |  |  |  |  |  | 2.1315 |  |
|  | | 2 |  | 36 |  | 50.972 |  |  |  |  |  | 1.2074 |  |
| MASC2 Self social anxiety |  | 1 |  | 16 |  | 58.13 |  |  |  |  |  | 1.8368 |  |
|  | | 2 |  | 36 |  | 52.111 |  |  |  |  |  | 1.1032 |  |
| MASC2 Self humil/reject |  | 1 |  | 16 |  | 53.44 |  |  |  |  |  | 1.8165 |  |
|  | | 2 |  | 36 |  | 48.444 |  |  |  |  |  | 0.9860 |  |
| **MASC Self performance fears** |  | **1** |  | 16 |  | **61.94** |  |  |  |  |  | 2.3192 |  |
|  | | 2 |  | 36 |  | 56.333 |  |  |  |  |  | 1.2078 |  |
| MASC2 Self obs/comp |  | 1 |  | 16 |  | 53.94 |  |  |  |  |  | 2.0645 |  |
|  | | 2 |  | 36 |  | 51.194 |  |  |  |  |  | 1.5165 |  |
| MASC2 Self physical symptoms |  | 1 |  | 16 |  | 57.63 |  |  |  |  |  | 2.2228 |  |
|  | | 2 |  | 36 |  | 51.278 |  |  |  |  |  | 1.2023 |  |
| MASC2 Self panic |  | 1 |  | 16 |  | 54.75 |  |  |  |  |  | 2.3354 |  |
|  | | 2 |  | 36 |  | 49.667 |  |  |  |  |  | 1.0676 |  |
| MASC2 Self tense/restless |  | 1 |  | 16 |  | 58.50 |  |  |  |  |  | 2.1311 |  |
|  | | 2 |  | 36 |  | 51.861 |  |  |  |  |  | 1.2843 |  |
| MASC2 Self avoidance |  | 1 |  | 16 |  | 53.13 |  |  |  |  |  | 1.0036 |  |
|  | | 2 |  | 36 |  | 51.556 |  |  |  |  |  | 0.7913 |  |
| MASC2 Self anxiety probab. |  | 1 |  | 16 |  | 1.38 |  |  |  |  |  | 0.2213 |  |
|  | | 2 |  | 36 |  | 0.917 |  |  |  |  |  | 0.1283 |  |
| MASC2 Self Inc. index. |  | 1 |  | 16 |  | 5.63 |  |  |  |  |  | 0.3400 |  |
|  | | 2 |  | 36 |  | 4.722 |  |  |  |  |  | 0.2806 |  |
| **MASC2 Parent Tot** |  | 1 |  | 16 |  | **62.13** |  |  |  |  |  | 2.7124 |  |
|  | | 2 |  | 36 |  | **61.250** |  |  |  |  |  | 2.0715 |  |
| MASC2 Parent sep anxiety/phobias |  | 1 |  | 16 |  | 58.31 |  |  |  |  |  | 2.4945 |  |
|  | | 2 |  | 36 |  | 59.639 |  |  |  |  |  | 1.8611 |  |
| **MASC2 Parent GAD** |  | 1 |  | 16 |  | **66.06** |  |  |  |  |  | 1.9028 |  |
|  | | 2 |  | 36 |  | **64.111** |  |  |  |  |  | 2.0412 |  |
| **MASC2 Parent social anxiety** |  | 1 |  | 16 |  | **69.38** |  |  |  |  |  | 2.2673 |  |
|  | | 2 |  | 36 |  | **63.778** |  |  |  |  |  | 2.8986 |  |
| MASC2 Parent humil/reject |  | 1 |  | 16 |  | 65.63 |  |  |  |  |  | 2.3960 |  |
|  | | 2 |  | 36 |  | 60.889 |  |  |  |  |  | 2.5090 |  |
| **MASC2 Parent performance fear** |  | 1 |  | 16 |  | **65.44** |  |  |  |  |  | 2.4477 |  |
|  | | 2 |  | 36 |  | **65.667** |  |  |  |  |  | 2.0931 |  |
| MASC2 Parent obs/comp |  | 1 |  | 16 |  | 48.13 |  |  |  |  |  | 2.4029 |  |
|  | | 2 |  | 36 |  | 49.500 |  |  |  |  |  | 2.1204 |  |
| MASC2 Parent physical symptoms* |  | 1 |  | 16 |  | 61.75 |  |  |  |  |  | 3.2178 |  |
|  | | 2 |  | 36 |  | 58.139 |  |  |  |  |  | 1.9141 |  |
| MASC2 Parent panic |  | 1 |  | 16 |  | 58.75 |  |  |  |  |  | 3.8574 |  |
|  | | 2 |  | 36 |  | 56.694 |  |  |  |  |  | 2.2342 |  |
| MASC2 Parent tense/restless |  | 1 |  | 16 |  | 61.94 |  |  |  |  |  | 2.4974 |  |
|  | | 2 |  | 36 |  | 58.056 |  |  |  |  |  | 1.4992 |  |
| MASC2 Parent avoidance |  | 1 |  | 16 |  | 53.50 |  |  |  |  |  | 1.7393 |  |
|  | | 2 |  | 36 |  | 57.278 |  |  |  |  |  | 1.3942 |  |
| MASC2 Parent anxiety probab. |  | 1 |  | 16 |  | 2.50 |  |  |  |  |  | 0.1581 |  |
|  | | 2 |  | 36 |  | 3.750 |  |  |  |  |  | 1.6185 |  |
| MASC2 Parent. Inc Index |  | 1 |  | 16 |  | 6.25 |  |  |  |  |  | 0.4425 |  |
|  | | 2 |  | 36 |  | 5.611 |  |  |  |  |  | 0.3806 |  |
| CDI2 Self Tot |  | 1 |  | 16 |  | 51.06 |  |  |  |  |  | 1.6367 |  |
|  | | 2 |  | 36 |  | 48.389 |  |  |  |  |  | 0.9430 |  |
| CDI2 Self Emot issues |  | 1 |  | 16 |  | 50.00 |  |  |  |  |  | 2.5199 |  |
|  | | 2 |  | 36 |  | 45.333 |  |  |  |  |  | 1.5487 |  |
| CDI2 Self neg mood/somatic sympt |  | 1 |  | 16 |  | 51.63 |  |  |  |  |  | 2.1367 |  |
|  | | 2 |  | 36 |  | 48.417 |  |  |  |  |  | 0.9987 |  |
| CDI2 Self neg self-esteem |  | 1 |  | 16 |  | 50.44 |  |  |  |  |  | 1.9126 |  |
|  | | 2 |  | 36 |  | 46.833 |  |  |  |  |  | 0.7448 |  |
| CDI2 Self functional imp |  | 1 |  | 16 |  | 55.50 |  |  |  |  |  | 1.5785 |  |
|  | | 2 |  | 36 |  | 53.722 |  |  |  |  |  | 1.1482 |  |
| CDI2 Self ineffect |  | 1 |  | 16 |  | 51.13 |  |  |  |  |  | 1.2906 |  |
|  | | 2 |  | 36 |  | 49.861 |  |  |  |  |  | 1.0706 |  |
| CDI2 Self interpers probl |  | 1 |  | 16 |  | 53.19 |  |  |  |  |  | 1.0925 |  |
|  | | 2 |  | 36 |  | 52.667 |  |  |  |  |  | 0.9751 |  |
| CDI2 Parent Tot |  | 1 |  | 16 |  | 60.06 |  |  |  |  |  | 3.3360 |  |
|  | | 2 |  | 36 |  | 55.111 |  |  |  |  |  | 1.4419 |  |
| CDI2 Parent Emot issues |  | 1 |  | 16 |  | 59.81 |  |  |  |  |  | 3.7095 |  |
|  | | 2 |  | 36 |  | 55.694 |  |  |  |  |  | 1.7843 |  |
| CDI2 Parent functional imp |  | 1 |  | 16 |  | 57.75 |  |  |  |  |  | 2.2164 |  |
|  | | 2 |  | 36 |  | 53.806 |  |  |  |  |  | 1.1040 |  |
| CPRS A |  | 1 |  | 16 |  | 57.63 |  |  |  |  |  | 3.0494 |  |
|  | | 2 |  | 36 |  | 56.167 |  |  |  |  |  | 1.4728 |  |
| CPRS B* |  | 1 |  | 16 |  | 68.50 | * |  |  |  |  | 3.3053 |  |
|  | | 2 |  | 36 |  | 63.944 | * |  |  |  |  | 1.7215 |  |
| CPRS C |  | 1 |  | 16 |  | 57.31 |  |  |  |  |  | 3.1685 |  |
|  | | 2 |  | 36 |  | 53.028 |  |  |  |  |  | 1.1585 |  |
| CPRS D* |  | 1 |  | 16 |  | 63.38 | * |  |  |  |  | 4.3088 |  |
|  | | 2 |  | 36 |  | 61.056 | * |  |  |  |  | 1.9036 |  |
| CPRS E |  | 1 |  | 16 |  | 54.94 |  |  |  |  |  | 4.8421 |  |
|  | | 2 |  | 36 |  | 56.028 |  |  |  |  |  | 1.8778 |  |
| CPRS F* |  | 1 |  | 16 |  | 69.63 | * |  |  |  |  | 3.8056 |  |
|  | | 2 |  | 36 |  | 65.389 | * |  |  |  |  | 2.3232 |  |
| CPRS G* |  | 1 |  | 16 |  | 61.81 | * |  |  |  |  | 2.5968 |  |
|  | | 2 |  | 36 |  | 61.056 | * |  |  |  |  | 2.0280 |  |
| CPRS H* |  | 1 |  | 16 |  | 64.06 | * |  |  |  |  | 2.8379 |  |
|  | | 2 |  | 36 |  | 62.833 | * |  |  |  |  | 1.9030 |  |
| CPRS I* |  | 1 |  | 16 |  | 58.88 |  |  |  |  |  | 2.7897 |  |
|  | | 2 |  | 36 |  | 55.861 |  |  |  |  |  | 1.3082 |  |
| CPRS J |  | 1 |  | 16 |  | 60.38 |  |  |  |  |  | 2.2618 |  |
|  | | 2 |  | 36 |  | 59.750 |  |  |  |  |  | 1.4239 |  |
| CPRS K |  | 1 |  | 16 |  | 59.69 |  |  |  |  |  | 2.8806 |  |
|  | | 2 |  | 36 |  | 56.528 |  |  |  |  |  | 1.2820 |  |
| CPRS L* |  | 1 |  | 16 |  | 65.69 | * |  |  |  |  | 2.6532 |  |
|  | | 2 |  | 36 |  | 61.472 | * |  |  |  |  | 1.6731 |  |
| CPRS M |  | 1 |  | 16 |  | 55.06 |  |  |  |  |  | 2.8144 |  |
|  | | 2 |  | 36 |  | 52.250 |  |  |  |  |  | 1.1625 |  |
| CPRS N* |  | 1 |  | 16 |  | 62.44 | * |  |  |  |  | 2.7688 |  |
|  | | 2 |  | 36 |  | 56.833 |  |  |  |  |  | 1.4285 |  |
| PSI- PD_Mother |  | 1 |  | 16 |  | 41.44 |  |  |  |  |  | 6.5390 |  |
|  | | 2 |  | 36 |  | 41.444 |  |  |  |  |  | 3.7179 |  |
| PSI-P-CDI_Mother* |  | 1 |  | 16 |  | 63.56 | * |  |  |  |  | 7.7819 |  |
|  | | 2 |  | 36 |  | 55.722 |  |  |  |  |  | 3.9590 |  |
| PSI- DC_Mother* |  | 1 |  | 16 |  | 62.50 | * |  |  |  |  | 7.3802 |  |
|  | | 2 |  | 36 |  | 53.611 |  |  |  |  |  | 4.1274 |  |
| PSI- Diff Resp_Mother |  | 1 |  | 16 |  | 59.88 |  |  |  |  |  | 7.8294 |  |
|  | | 2 |  | 36 |  | 46.750 |  |  |  |  |  | 3.8086 |  |
| PSI-Stress_Mother |  | 1 |  | 16 |  | 50.44 |  |  |  |  |  | 7.3104 |  |
|  | | 2 |  | 36 |  | 59.694 |  |  |  |  |  | 3.3808 |  |
| PSI-PD_Father |  | 1 |  | 16 |  | 50.75 |  |  |  |  |  | 3.8259 |  |
|  | | 2 |  | 36 |  | 50.083 |  |  |  |  |  | 2.6984 |  |
| PSI- P-CDI_ Father |  | 1 |  | 16 |  | 54.00 |  |  |  |  |  | 4.7496 |  |
|  | | 2 |  | 36 |  | 55.861 |  |  |  |  |  | 2.6554 |  |
| PSI- DC_Father |  | 1 |  | 16 |  | 54.13 |  |  |  |  |  | 5.1663 |  |
|  | | 2 |  | 36 |  | 53.111 |  |  |  |  |  | 2.8024 |  |
| PSI- Diff Resp_Father |  | 1 |  | 16 |  | 51.69 |  |  |  |  |  | 5.0972 |  |
|  | | 2 |  | 36 |  | 51.306 |  |  |  |  |  | 2.7854 |  |
| PSI-Stress_ Father |  | 1 |  | 16 |  | 53.50 |  |  |  |  |  | 4.1503 |  |
|  | | 2 |  | 36 |  | 53.306 |  |  |  |  |  | 2.0888 |  |
| C-GAS |  | 1 |  | 16 |  | 54.38 |  |  |  |  |  | 0.9214 |  |
|  | | 2 |  | 36 |  | 56.861 |  |  |  |  |  | 0.5789 |  |
| **SDSC DIS** |  | **1** |  | 16 |  | **74.75** |  |  |  |  |  | 3.4958 |  |
|  | | 2 |  | 36 |  | 55.833 |  |  |  |  |  | 1.9426 |  |
| SDSC DMS* |  | 1 |  | 16 |  | 64.00 | * |  |  |  |  | 4.8175 |  |
|  | | 2 |  | 36 |  | 49.806 |  |  |  |  |  | 1.1407 |  |
| SDSC SBD* |  | 1 |  | 16 |  | 62.56 | * |  |  |  |  | 4.4375 |  |
|  | | 2 |  | 36 |  | 48.111 |  |  |  |  |  | 0.5866 |  |
| SDSC SWTD |  | 1 |  | 16 |  | 59.94 |  |  |  |  |  | 3.3646 |  |
|  | | 2 |  | 36 |  | 48.333 |  |  |  |  |  | 1.0787 |  |
| SDSC DOES* |  | 1 |  | 16 |  | 66.38 | * |  |  |  |  | 3.6888 |  |
|  | | 2 |  | 36 |  | 52.250 |  |  |  |  |  | 1.7322 |  |
| SDSC SHY |  | 1 |  | 16 |  | 51.13 |  |  |  |  |  | 3.3026 |  |
|  | | 2 |  | 36 |  | 47.944 |  |  |  |  |  | 1.2071 |  |
|  | |  |  |  |  |  |  |  |  |  |  |  |  |
|  | | | | | | | | | | | | | |

*Legend.* M=mean;SE= standard error. Group 1= with sleep problems ad depicted by SDSC test; Group 2 = without sleep problems ad depicted by SDSC test. Marked with the mean of borderline scores. In marked black the mean of clinically significative scores.
