# Supplementary material for: Investigating the relationship between sleep disturbances and psychopathology In children and adolescents with microdeletion of 22q11 chromosome: an exploratory study
Source: Front Psychiatry. 2025 Jul 23;16:1595492. doi: 10.3389/fpsyt.2025.1595492 (PMC12325377; doi:10.3389/fpsyt.2025.1595492)
Supplement: Supplementary file 2 [file Table2.docx]

**Table 2 S. independent two-sample *t* test. Differences Between Goup 1 and Group 2 Scores on All Outcome Measures.**

|  | | **Statistic** | | | **df** | | **p** | | **Mean Difference** | | | **SE** | | **Cohen’s d+** | | |  |  |
| --- | --- | --- | --- | --- | --- | --- | --- | --- | --- | --- | --- | --- | --- | --- | --- | --- | --- | --- |
| FSQI |  |  | 0.5227 |  | | 50.0 |  | 0.603 |  | 3.55556 |  | | 6.8021 |  |  | 0.15706 | |  |
| WISC-IV VCI |  |  | 0.2801 |  | | 50.0 |  | 0.781 |  | 1.29167 |  | | 4.6116 |  |  | 0.08416 | |  |
| WISC-IV PRI |  |  | -0.0966 | ᵃ | | 50.0 |  | 0.923 |  | -0.45139 |  | | 4.6721 |  |  | -0.02903 | |  |
| WISC-IV WMI |  |  | 0.4727 |  | | 50.0 |  | 0.638 |  | 1.82639 |  | | 3.8638 |  |  | 0.14203 | |  |
| WISC IV PSI |  |  | -0.1275 |  | | 50.0 |  | 0.899 |  | -0.56250 |  | | 4.4108 |  |  | -0.03832 | |  |
| ABAS-GAC COMP |  |  | -0.8111 |  | | 50.0 |  | 0.421 |  | -4.58333 |  | | 5.6505 |  |  | -0.24372 | |  |
| ABAS-DAC COMP |  |  | -1.2310 |  | | 50.0 |  | 0.224 |  | -6.40972 |  | | 5.2071 |  |  | -0.36986 | |  |
| ABAS-DAS COMP |  |  | 0.2901 |  | | 50.0 |  | 0.773 |  | 1.81250 |  | | 6.2471 |  |  | 0.08717 | |  |
| ABAS-DAP COMP |  |  | -0.8793 |  | | 50.0 |  | 0.383 |  | -4.78472 |  | | 5.4416 |  |  | -0.26419 | |  |
| ABAS-GAC PERC |  |  | -0.8533 |  | | 50.0 |  | 0.398 |  | -6.36806 |  | | 7.4633 |  |  | -0.25637 | |  |
| ABAS-DAC PERC |  |  | -1.1617 |  | | 50.0 |  | 0.251 |  | -8.36806 |  | | 7.2032 |  |  | -0.34905 | |  |
| ABAS-DAS PERC |  |  | 0.0111 |  | | 50.0 |  | 0.991 |  | 0.08333 |  | | 7.5124 |  |  | 0.00333 | |  |
| ABAS-DAP PERC |  |  | -0.7952 |  | | 50.0 |  | 0.430 |  | -5.67917 |  | | 7.1416 |  |  | -0.23894 | |  |
| MASC2_SELF_TOT |  |  | 2.4371 |  | | 50.0 |  | 0.018 |  | 6.13889 |  | | 2.5190 |  |  | 0.73224 | |  |
| MASC2_SELF_ SELF SEP ANXIETY/PHOBIAS |  |  | 1.9321 |  | | 50.0 |  | 0.059 |  | 4.60417 |  | | 2.3829 |  |  | 0.58054 | |  |
| MASC2_SELF_GAD |  |  | 2.5403 |  | | 50.0 |  | 0.014 |  | 5.84028 |  | | 2.2990 |  |  | 0.76327 | |  |
| MASC2_ SELF SOCIAL ANXIETY |  |  | 2.9237 |  | | 50.0 |  | 0.005 |  | 6.01389 |  | | 2.0569 |  |  | 0.87847 | |  |
| MASC2_ SELF HUMIL/REJECT |  |  | 2.6165 |  | | 50.0 |  | 0.012 |  | 4.99306 |  | | 1.9083 |  |  | 0.78617 | |  |
| MASC SELF PERFORMANCE FEARS |  |  | 2.3578 |  | | 50.0 |  | 0.022 |  | 5.60417 |  | | 2.3769 |  |  | 0.70843 | |  |
| MASC2 SELF OBS/COMP |  |  | 1.0310 |  | | 50.0 |  | 0.308 |  | 2.74306 |  | | 2.6606 |  |  | 0.30977 | |  |
| MASC2 SELF PHYSICAL SYMPTOMS |  |  | 2.7240 |  | | 50.0 |  | 0.009 |  | 6.34722 |  | | 2.3301 |  |  | 0.81847 | |  |
| MASC2 SELF PANIC |  |  | 2.2833 |  | | 50.0 |  | 0.027 |  | 5.08333 |  | | 2.2263 |  |  | 0.68606 | |  |
| MASC2 SELF TENSE/RESTLESS |  |  | 2.7757 |  | | 50.0 |  | 0.008 |  | 6.63889 |  | | 2.3918 |  |  | 0.83400 | |  |
| MASC2 SELF AVOIDANCE |  |  | 1.1505 |  | | 50.0 |  | 0.255 |  | 1.56944 |  | | 1.3641 |  |  | 0.34568 | |  |
| MASC2 SELF ANXIETY PROBAB |  |  | 1.8921 |  | | 50.0 |  | 0.064 |  | 0.45833 |  | | 0.2422 |  |  | 0.56849 | |  |
| MASC2 SELF INC. INDEX. |  |  | 1.8855 |  | | 50.0 |  | 0.065 |  | 0.90278 |  | | 0.4788 |  |  | 0.56652 | |  |
| MASC2_PARENT_TOT |  |  | 0.2431 |  | | 50.0 |  | 0.809 |  | 0.87500 |  | | 3.5987 |  |  | 0.07306 | |  |
| MASC2 PARENT SEP ANXIETY/PHOBIAS |  |  | -0.4078 |  | | 50.0 |  | 0.685 |  | -1.32639 |  | | 3.2522 |  |  | -0.12254 | |  |
| MASC2_PARENT_GAD |  |  | 0.5871 |  | | 50.0 |  | 0.560 |  | 1.95139 |  | | 3.3237 |  |  | 0.17640 | |  |
| MASC2_PARENT_ SOCIAL ANXIETY |  |  | 1.2116 | ᵃ | | 50.0 |  | 0.231 |  | 5.59722 |  | | 4.6197 |  |  | 0.36404 | |  |
| MASC2 PARENT HUMIL/REJECT |  |  | 1.1552 | ᵃ | | 50.0 |  | 0.253 |  | 4.73611 |  | | 4.0998 |  |  | 0.34709 | |  |
| MASC2 PARENT PERFORMANCE FEAR |  |  | -0.0647 |  | | 50.0 |  | 0.949 |  | -0.22917 |  | | 3.5444 |  |  | -0.01943 | |  |
| MASC2 PARENT OBS/COMP |  |  | -0.3854 |  | | 50.0 |  | 0.702 |  | -1.37500 |  | | 3.5680 |  |  | -0.11579 | |  |
| MASC2 PARENT PHYSICAL SYMPTOMS |  |  | 1.0085 |  | | 50.0 |  | 0.318 |  | 3.61111 |  | | 3.5808 |  |  | 0.30301 | |  |
| MASC2 PARENT PANIC |  |  | 0.4872 |  | | 50.0 |  | 0.628 |  | 2.05556 |  | | 4.2195 |  |  | 0.14637 | |  |
| MASC2 PARENT TENSE/RESTLESS |  |  | 1.3885 |  | | 50.0 |  | 0.171 |  | 3.88194 |  | | 2.7957 |  |  | 0.41720 | |  |
| MASC2 PARENT AVOIDANCE |  |  | -1.5778 |  | | 50.0 |  | 0.121 |  | -3.77778 |  | | 2.3944 |  |  | -0.47406 | |  |
| MASC2 PARENT ANXIETY PROBAB. |  |  | -0.5116 |  | | 50.0 |  | 0.611 |  | -1.25000 |  | | 2.4435 |  |  | -0.15371 | |  |
| MASC2 PARENT. INC INDEX |  |  | 0.9924 |  | | 50.0 |  | 0.326 |  | 0.63889 |  | | 0.6438 |  |  | 0.29817 | |  |
| CDI2_SELF_TOT |  |  | 1.4983 |  | | 50.0 |  | 0.140 |  | 2.67361 |  | | 1.7844 |  |  | 0.45019 | |  |
| CDI2 SELF EMOT ISSUES |  |  | 1.6289 |  | | 50.0 |  | 0.110 |  | 4.66667 |  | | 2.8650 |  |  | 0.48941 | |  |
| CDI2 SELF NEG MOOD/SOMATIC SYMPT |  |  | 1.5567 |  | | 50.0 |  | 0.126 |  | 3.20833 |  | | 2.0610 |  |  | 0.46773 | |  |
| CDI2 SELF NEG SELF-ESTEEM |  |  | 2.1360 |  | | 50.0 |  | 0.038 |  | 3.60417 |  | | 1.6874 |  |  | 0.64177 | |  |
| CDI2 SELF FUNCTIONAL IMP |  |  | 0.8802 |  | | 50.0 |  | 0.383 |  | 1.77778 |  | | 2.0197 |  |  | 0.26447 | |  |
| CDI2 SELF INEFFECT |  |  | 0.6927 |  | | 50.0 |  | 0.492 |  | 1.26389 |  | | 1.8247 |  |  | 0.20812 | |  |
| CDI2 SELF INTERPERS PROBL |  |  | 0.3181 |  | | 50.0 |  | 0.752 |  | 0.52083 |  | | 1.6371 |  |  | 0.09559 | |  |
| CDI2_PARENT_TOT |  |  | 1.6020 |  | | 50.0 |  | 0.115 |  | 4.95139 |  | | 3.0907 |  |  | 0.48135 | |  |
| CDI2 PARENT EMOT ISSUES |  |  | 1.1332 |  | | 50.0 |  | 0.263 |  | 4.11806 |  | | 3.6340 |  |  | 0.34048 | |  |
| CDI2 PARENT EMOT ISSUES |  |  | 1.7817 |  | | 50.0 |  | 0.081 |  | 3.94444 |  | | 2.2139 |  |  | 0.53532 | |  |
| CPRS A |  |  | 0.4871 |  | | 50.0 |  | 0.628 |  | 1.45833 |  | | 2.9940 |  |  | 0.14635 | |  |
| CPRS B |  |  | 1.3448 |  | | 50.0 |  | 0.185 |  | 4.55556 |  | | 3.3876 |  |  | 0.40405 | |  |
| CPRS C |  |  | 1.5747 | ᵃ | | 50.0 |  | 0.122 |  | 4.28472 |  | | 2.7209 |  |  | 0.47314 | |  |
| CPRS D |  |  | 0.5747 | ᵃ | | 50.0 |  | 0.568 |  | 2.31944 |  | | 4.0360 |  |  | 0.17267 | |  |
| CPRS E |  |  | -0.2557 |  | | 50.0 |  | 0.799 |  | -1.09028 |  | | 4.2640 |  |  | -0.07683 | |  |
| CPRS F |  |  | 0.9834 |  | | 50.0 |  | 0.330 |  | 4.23611 |  | | 4.3075 |  |  | 0.29548 | |  |
| CPRS G |  |  | 0.2160 |  | | 50.0 |  | 0.830 |  | 0.75694 |  | | 3.5041 |  |  | 0.06491 | |  |
| CPRS H |  |  | 0.3589 |  | | 50.0 |  | 0.721 |  | 1.22917 |  | | 3.4247 |  |  | 0.10784 | |  |
| CPRS I |  |  | 1.1181 |  | | 50.0 |  | 0.269 |  | 3.01389 |  | | 2.6955 |  |  | 0.33595 | |  |
| CPRS J |  |  | 0.2392 |  | | 50.0 |  | 0.812 |  | 0.62500 |  | | 2.6133 |  |  | 0.07186 | |  |
| CPRS K |  |  | 1.1667 |  | | 50.0 |  | 0.249 |  | 3.15972 |  | | 2.7083 |  |  | 0.35054 | |  |
| CPRS L |  |  | 1.3735 |  | | 50.0 |  | 0.176 |  | 4.21528 |  | | 3.0690 |  |  | 0.41269 | |  |
| CPRS M |  |  | 1.1026 | ᵃ | | 50.0 |  | 0.275 |  | 2.81250 |  | | 2.5508 |  |  | 0.33129 | |  |
| CPRS N |  |  | 1.9858 |  | | 50.0 |  | 0.053 |  | 5.60417 |  | | 2.8221 |  |  | 0.59667 | |  |
| PSI- PD_MOTHER |  |  | -9.82E−4 |  | | 50.0 |  | 0.999 |  | -0.00694 |  | | 7.0693 |  |  | -2.95E−4 | |  |
| PSI-P-CDI_MOTHER |  |  | 0.9965 |  | | 50.0 |  | 0.324 |  | 7.84028 |  | | 7.8677 |  |  | 0.29942 | |  |
| PSI- DC_MOTHER |  |  | 1.1256 |  | | 50.0 |  | 0.266 |  | 8.88889 |  | | 7.8967 |  |  | 0.33822 | |  |
| PSI- DIFF RESP_MOTHER |  |  | 1.7006 |  | | 50.0 |  | 0.095 |  | 13.12500 |  | | 7.7177 |  |  | 0.51098 | |  |
| PSI-STRESS_MOTHER |  |  | -1.3203 |  | | 50.0 |  | 0.193 |  | -9.25694 |  | | 7.0115 |  |  | -0.39669 | |  |
| PSI-PD_FATHER |  |  | 0.1393 |  | | 50.0 |  | 0.890 |  | 0.66667 |  | | 4.7863 |  |  | 0.04185 | |  |
| PSI- P-CDI_ FATHER |  |  | -0.3663 |  | | 50.0 |  | 0.716 |  | -1.86111 |  | | 5.0810 |  |  | -0.11006 | |  |
| PSI_DC_ FATHER |  |  | 0.1869 |  | | 50.0 |  | 0.853 |  | 1.01389 |  | | 5.4252 |  |  | 0.05615 | |  |
| PSI_RISP DIFF_ FATHER |  |  | 0.0710 |  | | 50.0 |  | 0.944 |  | 0.38194 |  | | 5.3767 |  |  | 0.02134 | |  |
| PSI_STRESS_ FATHER |  |  | 0.0466 |  | | 50.0 |  | 0.963 |  | 0.19444 |  | | 4.1702 |  |  | 0.01401 | |  |
| C-GAS |  |  | -2.3383 |  | | 50.0 |  | 0.023 |  | -2.48611 |  | | 1.0632 |  |  | -0.70259 | |  |
| K-MAJOR DEPRESSIVE DISORDER |  |  | -0.5913 |  | | 50.0 |  | 0.557 |  | -0.03472 |  | | 0.0587 |  |  | -0.17767 | |  |
| K-E.MAN |  |  | NAN | ᵇ | |  |  |  |  |  |  | |  |  |  |  | |  |
| K-PSYCHOTIC EPISODE |  |  | -0.4133 | ᵃ | | 50.0 |  | 0.681 |  | -0.03472 |  | | 0.0840 |  |  | -0.12417 | |  |
| K-PANIC DISORDER |  |  | NAN | ᵇ | |  |  |  |  |  |  | |  |  |  |  | |  |
| K-SEPARATION ANXIETY DISORDER |  |  | 1.5374 |  | | 50.0 |  | 0.130 |  | 0.09028 |  | | 0.0587 |  |  | 0.46193 | |  |
| K-SOCIAL PHOBIA (SOCIAL ANXIETY DISORDER) |  |  | 1.2646 |  | | 50.0 |  | 0.212 |  | 0.11806 |  | | 0.0934 |  |  | 0.37998 | |  |
| K-SPECIFIC PHOBIA |  |  | 1.5374 |  | | 50.0 |  | 0.130 |  | 0.09028 |  | | 0.0587 |  |  | 0.46193 | |  |
| K-GENERALIZED ANXIETY DISORDER (GAD) |  |  | 0.8133 |  | | 50.0 |  | 0.420 |  | 0.13194 |  | | 0.1622 |  |  | 0.24437 | |  |
| K-OBSESSIVE-COMPULSIVE DISORDER (OCD) |  |  | 0.3792 |  | | 50.0 |  | 0.706 |  | 0.02778 |  | | 0.0733 |  |  | 0.11393 | |  |
| K-NONORGANIC ENURESIS |  |  | NAN | ᵇ | |  |  |  |  |  |  | |  |  |  |  | |  |
| K-NONORGANIC ENCOPRESIS |  |  | NAN | ᵇ | |  |  |  |  |  |  | |  |  |  |  | |  |
| K-ANOREXIA NERVOSA |  |  | NAN | ᵇ | |  |  |  |  |  |  | |  |  |  |  | |  |
| K-BUL.NERV |  |  | NAN | ᵇ | |  |  |  |  |  |  | |  |  |  |  | |  |
| K-ATTENTION-DEFICIT/HYPERACTIVITY DISORDER |  |  | -1.1923 | ᵃ | | 50.0 |  | 0.239 |  | -0.17361 |  | | 0.1456 |  |  | -0.35824 | |  |
| K-OPPOSITIONAL DEFIANT DISORDER (ODD) |  |  | -1.5191 | ᵃ | | 50.0 |  | 0.135 |  | -0.12500 |  | | 0.0823 |  |  | -0.45644 | |  |
| K-CONDUCT DISORDER |  |  | NAN | ᵇ | |  |  |  |  |  |  | |  |  |  |  | |  |
| K-TIC DISORDER |  |  | NAN | ᵇ | |  |  |  |  |  |  | |  |  |  |  | |  |
| K-SUBSTANCE ABUSE |  |  | NAN | ᵇ | |  |  |  |  |  |  | |  |  |  |  | |  |
| K-POST-TRAUMATIC STRESS DISORDER (PTSD) |  |  | NAN | ᵇ | |  |  |  |  |  |  | |  |  |  |  | |  |
| K5-MOOD DYSREGULATION |  |  | 1.5191 | ᵃ | | 50.0 |  | 0.135 |  | 0.06250 |  | | 0.0411 |  |  | 0.45644 | |  |
| K5-AGORAPHOBIA |  |  | NAN | ᵇ | |  |  |  |  |  |  | |  |  |  |  | |  |
| K5-SELECTIVE MUTISM |  |  | NAN | ᵇ | |  |  |  |  |  |  | |  |  |  |  | |  |
| K5-AUTISM SPECTRUM DISORDER (ASD) |  |  | NAN | ᵇ | |  |  |  |  |  |  | |  |  |  |  | |  |
| K-ADHD_TYPE |  |  | -1.4365 |  | | 50.0 |  | 0.157 |  | -0.31944 |  | | 0.2224 |  |  | -0.43161 | |  |
| SDSC |  |  | 5.0773 |  | | 50.0 |  | < .001 |  | 18.91667 |  | | 3.7257 |  |  | 1.52554 | |  |
| SDSC DMS |  |  | 3.9342 | ᵃ | | 50.0 |  | < .001 |  | 14.19444 |  | | 3.6080 |  |  | 1.18208 | |  |
| SDSC SBD |  |  | 4.7348 | ᵃ | | 50.0 |  | < .001 |  | 14.45139 |  | | 3.0522 |  |  | 1.42263 | |  |
| SDSC SWTD |  |  | 4.2225 | ᵃ | | 50.0 |  | < .001 |  | 11.60417 |  | | 2.7482 |  |  | 1.26870 | |  |
| SDSC DOES |  |  | 3.9600 |  | | 50.0 |  | < .001 |  | 14.12500 |  | | 3.5669 |  |  | 1.18983 | |  |
| SDSC SHY |  |  | 1.1216 | ᵃ | | 50.0 |  | 0.267 |  | 3.18056 |  | | 2.8357 |  |  | 0.33700 | |  |
