# Supplementary material for: Investigating the relationship between sleep disturbances and psychopathology In children and adolescents with microdeletion of 22q11 chromosome: an exploratory study
Source: Front Psychiatry. 2025 Jul 23;16:1595492. doi: 10.3389/fpsyt.2025.1595492 (PMC12325377; doi:10.3389/fpsyt.2025.1595492)
Supplement: Supplementary file 3 [file Table3.docx]

**Table 3S: Demographic study of psychiatric diagnosis in the cohort by using K SADS**

**Group N Mean Standard error**

K-DEP 1 16 1.94 0.0625

2 36 1.972 0.0278

K-man.ep 1 16 2.00 0.0000

2 36 2.00 0.0000

K-psych.ep 1 16 1.94 0.1106

2 36 1.972 0.0278

K-PAD 1 16 2.00 0.0000

2 36 2.00 0.0000

K- sep anx 1 16 2.06 0.0625

2 36 1.972 0.0278

K- soc ph 1 16 2.06 0.1106

2 36 1.944 0.0387

K- spec ph 1 16 2.06 0.0625

2 36 1.972 0.0278

K-GAD 1 16 1.94 0.1434

2 36 1.806 0.0875

K-OCD 1 16 2.00 0.0000

2 36 1.972 0.0486

K-ENU 1 16 2.00 0.0000

2 36 2.00 0.0000

K-ENC 1 16 2.00 0.0000

2 36 2.00 0.0000

K-ANOR 1 16 2.00 0.0000

2 36 2.00 0.0000

K-BUL 1 16 2.00 0.0000

2 36 2.00 0.0000

K-ADHD 1 16 1.69 0.1505

2 36 1.861 0.0707

K-ODD 1 16 1.88 0.0854

2 36 2.00 0.0398

K-CD 1 16 2.00 0.0000

2 36 2.00 0.0000

K-TIC 1 16 2.00 0.0000

2 36 2.00 0.0000

K-subs.ab 1 16 2.00 0.0000

2 36 2.00 0.0000

K-PTSD 1 16 2.00 0.0000

2 36 2.00 0.0000

K5-mood disr 1 16 2.06 0.0625

2 36 2.00 0.0000

K5-agoraph 1 16 2.00 0.0000

2 36 2.00 0.0000

K5-sel mut 1 16 2.00 0.0000

2 36 2.00 0.0000

K5-ASD 1 16 2.00 0.0000

2 36 2.00 0.0000

Legend. M=mean;SE= standard error. Group 1= with sleep problems as depicted by SDSC test; Group 2 =without sleep problems as depicted by SDSC test.

**TABLE3S: Descriptive**

| \| **Disorders** \| \| **Group** \| \| **N** \| \| **Mean** \| \| **SE** \| \|  \| \| \| --- \| --- \| --- \| --- \| --- \| --- \| --- \| --- \| --- \| --- \| --- \| --- \| \| Eating D. \|  \| 1 \|  \| 16 \|  \| 2.00 \|  \| 0.0000 \|  \|  \|  \| \|  \|  \| 2 \|  \| 36 \|  \| 2.00 \|  \| 0.0000 \|  \|  \|  \| \| Neurodevelop \|  \| 1 \|  \| 16 \|  \| 2.00 \|  \| 0.0000 \|  \|  \|  \| \|  \|  \| 2 \|  \| 36 \|  \| 2.00 \|  \| 0.0000 \|  \|  \|  \| \| Mood \|  \| 1 \|  \| 16 \|  \| 1.88 \|  \| 0.0854 \|  \|  \|  \| \|  \|  \| 2 \|  \| 36 \|  \| 1.97 \|  \| 0.0278 \|  \|  \|  \| \| Anxiety \|  \| 1 \|  \| 16 \|  \| 1.44 \|  \| 0.1281 \|  \|  \|  \| \|  \|  \| 2 \|  \| 36 \|  \| 1.67 \|  \| 0.0797 \|  \|  \|  \| \| Behavior \|  \| 1 \|  \| 16 \|  \| 1.63 \|  \| 0.1250 \|  \|  \|  \| \|  \|  \| 2 \|  \| 36 \|  \| 1.78 \|  \| 0.0703 \|  \|  \|  \| \| Psychosis/OCD \|  \| 1 \|  \| 16 \|  \| 1.81 \|  \| 0.1008 \|  \|  \|  \| \|  \|  \| 2 \|  \| 36 \|  \| 1.89 \|  \| 0.0531 \|  \|  \|  \| \| Enco/Enur \|  \| 1 \|  \| 16 \|  \| 2.00 \|  \| 0.0000 \|  \|  \|  \| \|  \|  \| 2 \|  \| 36 \|  \| 2.00 \|  \| 0.0000 \|  \|  \|  \|    Legend. M=mean;SE= standard error. Group 1= with sleep problems as depicted by SDSC test; Group 2 =without sleep problems as depicted by SDSC test. |
| --- | --- | --- | --- | --- | --- | --- | --- | --- | --- | --- | --- | --- | --- | --- | --- | --- | --- | --- | --- | --- | --- | --- | --- | --- | --- | --- | --- | --- | --- | --- | --- | --- | --- | --- | --- | --- | --- | --- | --- | --- | --- | --- | --- | --- | --- | --- | --- | --- | --- | --- | --- | --- | --- | --- | --- | --- | --- | --- | --- | --- | --- | --- | --- | --- | --- | --- | --- | --- | --- | --- | --- | --- | --- | --- | --- | --- | --- | --- | --- | --- | --- | --- | --- | --- | --- | --- | --- | --- | --- | --- | --- | --- | --- | --- | --- | --- | --- | --- | --- | --- | --- | --- | --- | --- | --- | --- | --- | --- | --- | --- | --- | --- | --- | --- | --- | --- | --- | --- | --- | --- | --- | --- | --- | --- | --- | --- | --- | --- | --- | --- | --- | --- | --- | --- | --- | --- | --- | --- | --- | --- | --- | --- | --- | --- | --- | --- | --- | --- | --- | --- | --- | --- | --- | --- | --- | --- | --- | --- | --- | --- | --- | --- | --- | --- | --- | --- | --- | --- | --- | --- | --- | --- | --- | --- | --- | --- | --- | --- | --- | --- |

**Table 3S. Group differences in the frequencies of diagnoses between groups.**

| **Table 3S A**. Contingency Table - Mood disorders | | | | | | | |
| --- | --- | --- | --- | --- | --- | --- | --- |
|  | | **Mood** | | | |  | |
| **Group** | | **1** | | **2** | | **Tot** | |
| 1 |  | 2 |  | 14 |  | 16 |  |
| 2 |  | 1 |  | 35 |  | 36 |  |
| Tot |  | 3 |  | 49 |  | 52 |  |
|  | | | | | | | |

| χ² Test | | | | | | | |
| --- | --- | --- | --- | --- | --- | --- | --- |
|  | | **Value** | | **df** | | **p** | |
| χ² |  | 1.93 |  | 1 |  | 0.165 |  |
| N |  | 52 |  |  | |  | |
|  | | | | | | | |

| **Table 3S B.** Contingency Table – Anxiety disorders | | | | | | | |
| --- | --- | --- | --- | --- | --- | --- | --- |
|  | | **Anxiety** | | | |  | |
| **Group** | | **1** | | **2** | | **Tot** | |
| 1 |  | 9 |  | 7 |  | 16 |  |
| 2 |  | 12 |  | 24 |  | 36 |  |
| Tot |  | 21 |  | 31 |  | 52 |  |
|  | | | | | | | |

| χ² Test | | | | | | | |
| --- | --- | --- | --- | --- | --- | --- | --- |
|  | | **Value** | | **df** | | **p** | |
| χ² |  | 2.42 |  | 1 |  | 0.120 |  |
| N |  | 52 |  |  | |  | |
|  | | | | | | | |

| **Table 3S C.** Contingency Table – Behavioral disorders | | | | | | | |
| --- | --- | --- | --- | --- | --- | --- | --- |
|  | | **Behavioral** | | | |  | |
| **Group** | | **1** | | **2** | | **Tot** | |
| 1 |  | 6 |  | 10 |  | 16 |  |
| 2 |  | 8 |  | 28 |  | 36 |  |
| Tot |  | 14 |  | 38 |  | 52 |  |
|  | | | | | | | |

| χ² Test | | | | | | | |
| --- | --- | --- | --- | --- | --- | --- | --- |
|  | | **Value** | |  | | **p** | |
| χ² |  | 1.31 |  | 1 |  | 0.252 |  |
| N |  | 52 |  |  | |  | |
|  | | | | | | | |

| **Table 3S C.** Contingency Table – Psychosis/OCD disorders | | | | | | | |
| --- | --- | --- | --- | --- | --- | --- | --- |
|  | | **Psychosis/OCD** | | | |  | |
| **Group** | | **1** | | **2** | | **Tot** | |
| 1 |  | 3 |  | 13 |  | 16 |  |
| 2 |  | 4 |  | 32 |  | 36 |  |
| Tot |  | 7 |  | 45 |  | 52 |  |

| χ² Test | | | | | | | |
| --- | --- | --- | --- | --- | --- | --- | --- |
|  | | **Value** | | **df** | | **p** | |
| χ² |  | 0.555 |  | 1 |  | 0.456 |  |
| N |  | 52 |  |  | |  | |
|  | | | | | | | |

|  |
| --- |

|  |
| --- |
